# Supplementary figures and images for: Connecting basal body and mitochondrial DNA: TAC53 and the tubular organization of the tripartite attachment complex
Source: PLoS Pathog. 2025 Sep 15;21(9):e1013521. doi: 10.1371/journal.ppat.1013521 (PMC12453217; doi:10.1371/journal.ppat.1013521)

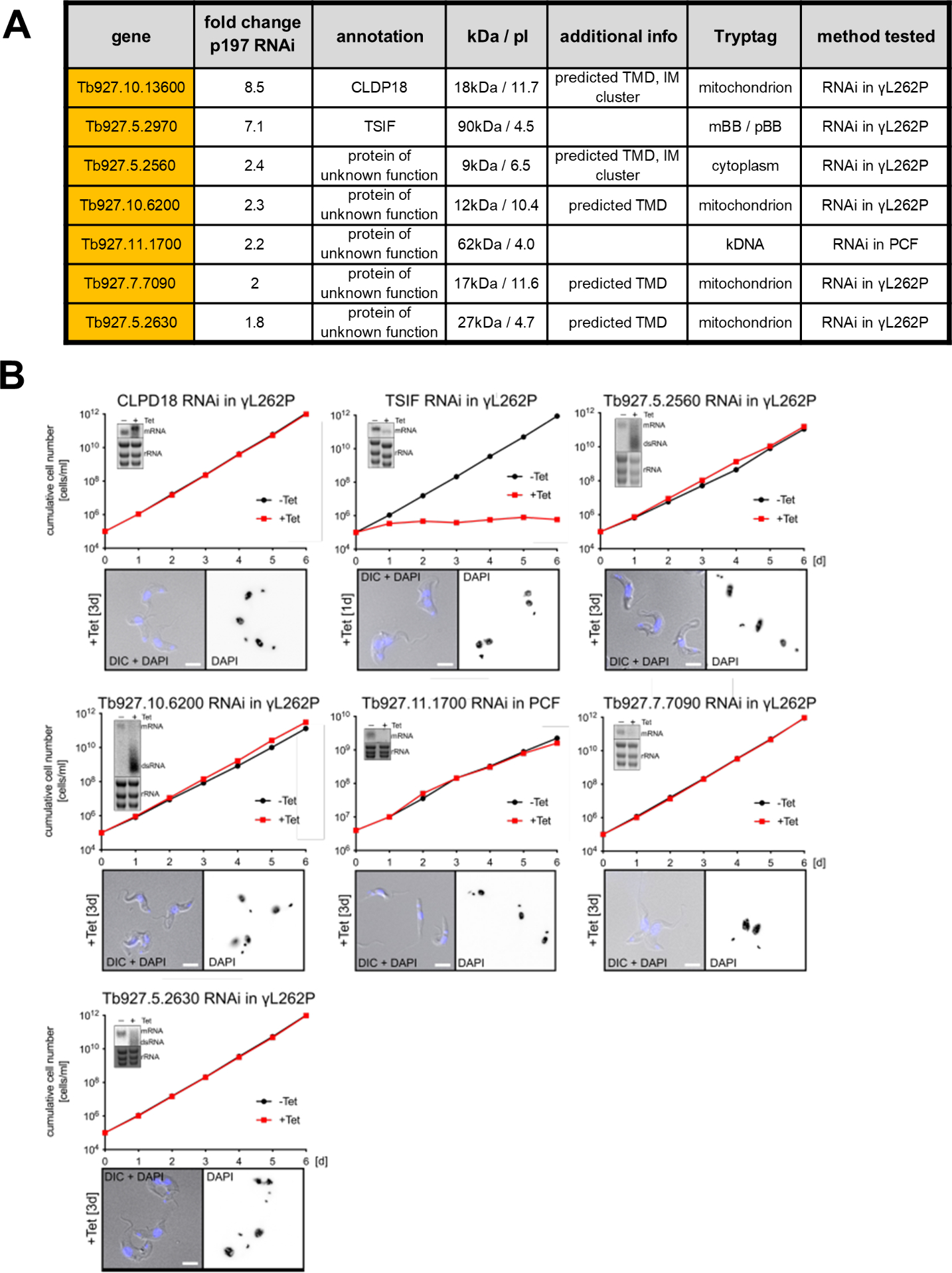

Supplement: S1 Fig — (A) Table of candidate proteins depleted by more than 1.5-fold upon p197-RNAi induction. The table includes accession number, fold change, annotation, molecular weight (kDa), isoelectric point (pI), predicted domains, their localization according to TrypTag.org, and the tested method. (B) Growth curves (GC, upper panel) over 6 days for uninduced (-Tet, black) and RNAi-induced (+Tet, red) cells, along with DAPI stainings (lower panel) of the corresponding tested candidates. The tested gene and cell type are indicated above the GC. Time points of DAPI stainings are marked on the left of the images. GC inset: RNAi efficiency was tested using a northern blot (NB) probed against the respective RNAi target. Ethidium bromide-stained rRNAs serve as the loading control. Scale bar is 5 μm. (TIF) [file ppat.1013521.s001.tif]

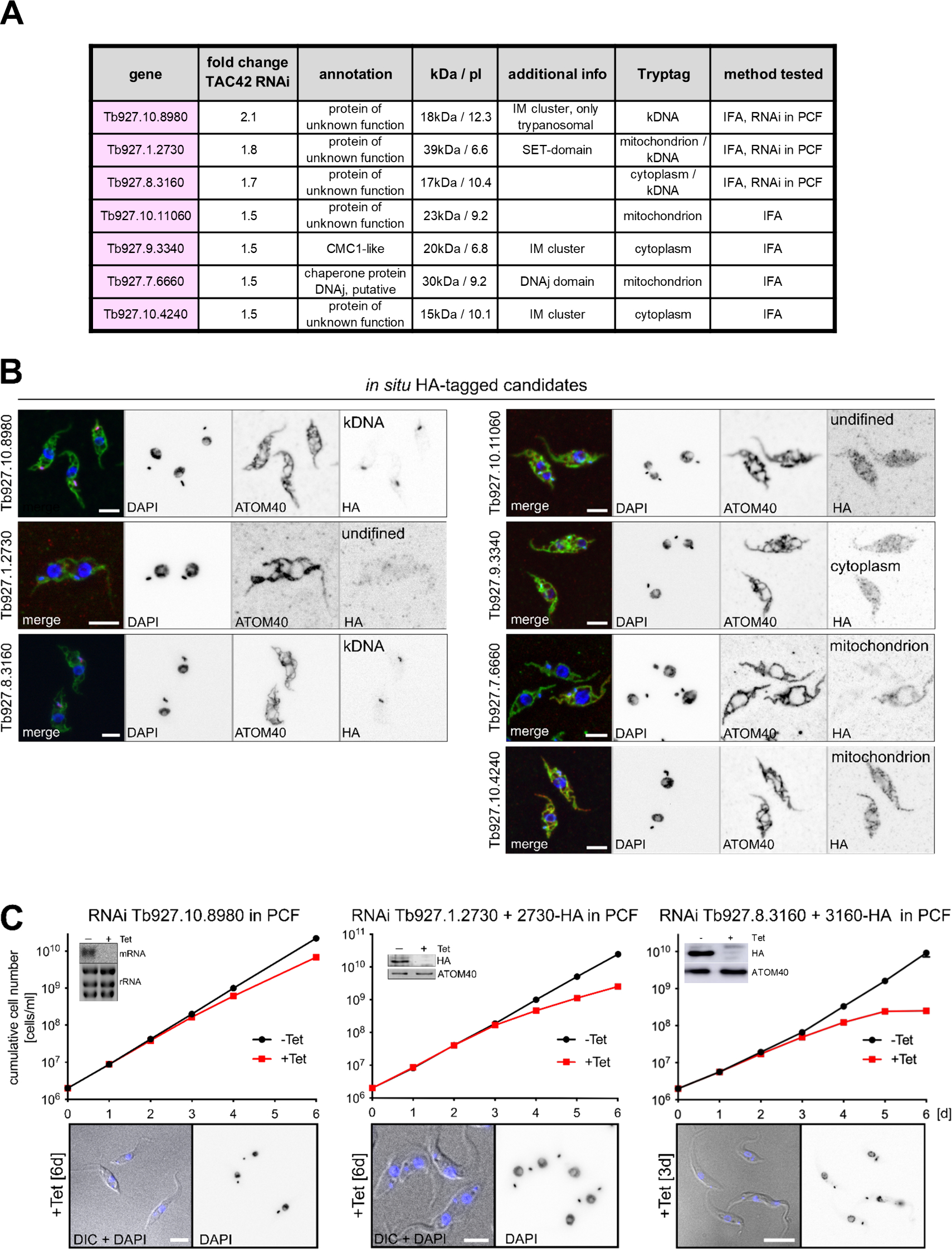

Supplement: S2 Fig — (A) Table of candidate proteins depleted by more than 1.5-fold upon TAC42-RNAi induction. The table includes accession number, fold change, annotation, molecular weight (kDa), isoelectric point (pI), predicted domains, their localization according to TrypTag.org, and the tested method. (B) Immunofluorescence images showing the localization of the respective candidate genes. Cells were stained with DAPI (blue), anti-ATOM40 (green) as a mitochondrial marker, and anti-HA (red) for the in-situ HA-tagged candidate proteins. Localization patterns are indicated in the images showing the HA staining. Scale bar: 5 μm. (C) Growth curves (upper panel) over 6 days of uninduced (-Tet, black) and RNAi-induced (+Tet, red) cells, along with DAPI stainings (lower panel) of the corresponding tested candidates. The tested gene and cell type are indicated above the growth curve. Time points of DAPI stainings are marked on the left of the images. Growth curve left inset: RNAi efficiency was tested using a northern blot (NB) probed against the respective RNAi target. Ethidium bromide-stained rRNAs serve as the loading control. Growth curve middle and right inset: RNAi efficiency has been tested using a WB probed against the RNAi target and ATOM40 as loading control. Scale bar is 5 μm. (TIF) [file ppat.1013521.s002.tif]

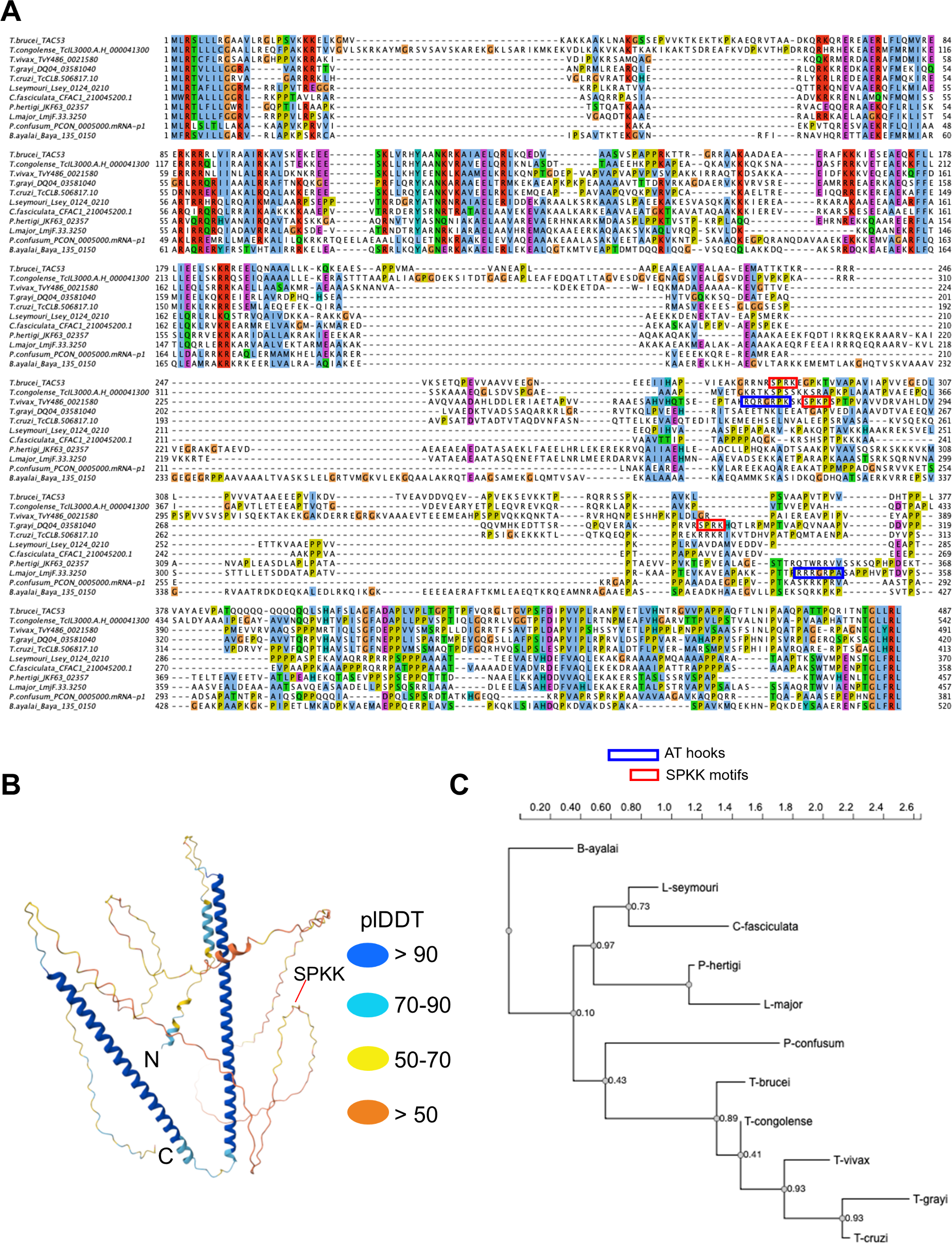

Supplement: S3 Fig — (A) Multiple Sequence Alignment of TAC53 Homologs in Trypanosomatids. The alignment highlights the presence of AT-hooks and SPKK motifs in some TAC53 homologs. AT-hooks are shown in blue, while SPKK motifs are shown in red. (B) AlphaFold-predicted structure of TAC53. The N- and C-termini are indicated, and the location of the SPKK motif is highlighted. The model is colored according to the predicted local distance difference test (pLDDT) confidence scores, with the corresponding color scale. (C) S3 Phylogenetic reconstruction with branchpoint support of representative sequences of TAC53. (TIF) [file ppat.1013521.s003.tif]

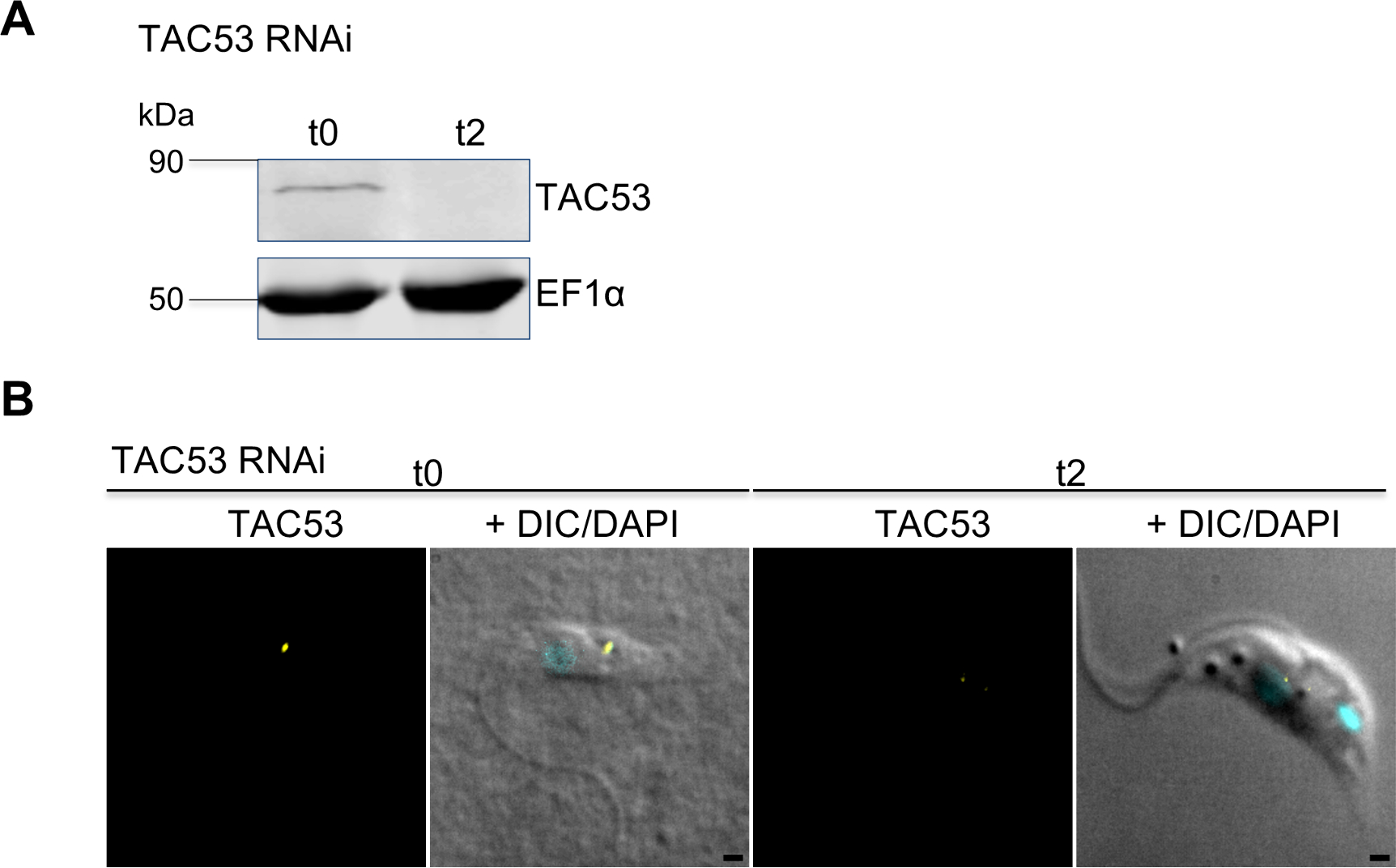

Supplement: S4 Fig — (A) Western blot showing TAC53 signal in RNAi-uninduced and induced cells [2d]. EF1α is used as a loading control. (B) Immunofluorescence assay showing TAC53 signal in RNAi-uninduced and induced cells [2d]. TAC53 antibody signal is shown in yellow, and DAPI staining is shown in cyan. Scale bar is 1 μm. (TIF) [file ppat.1013521.s004.tif]

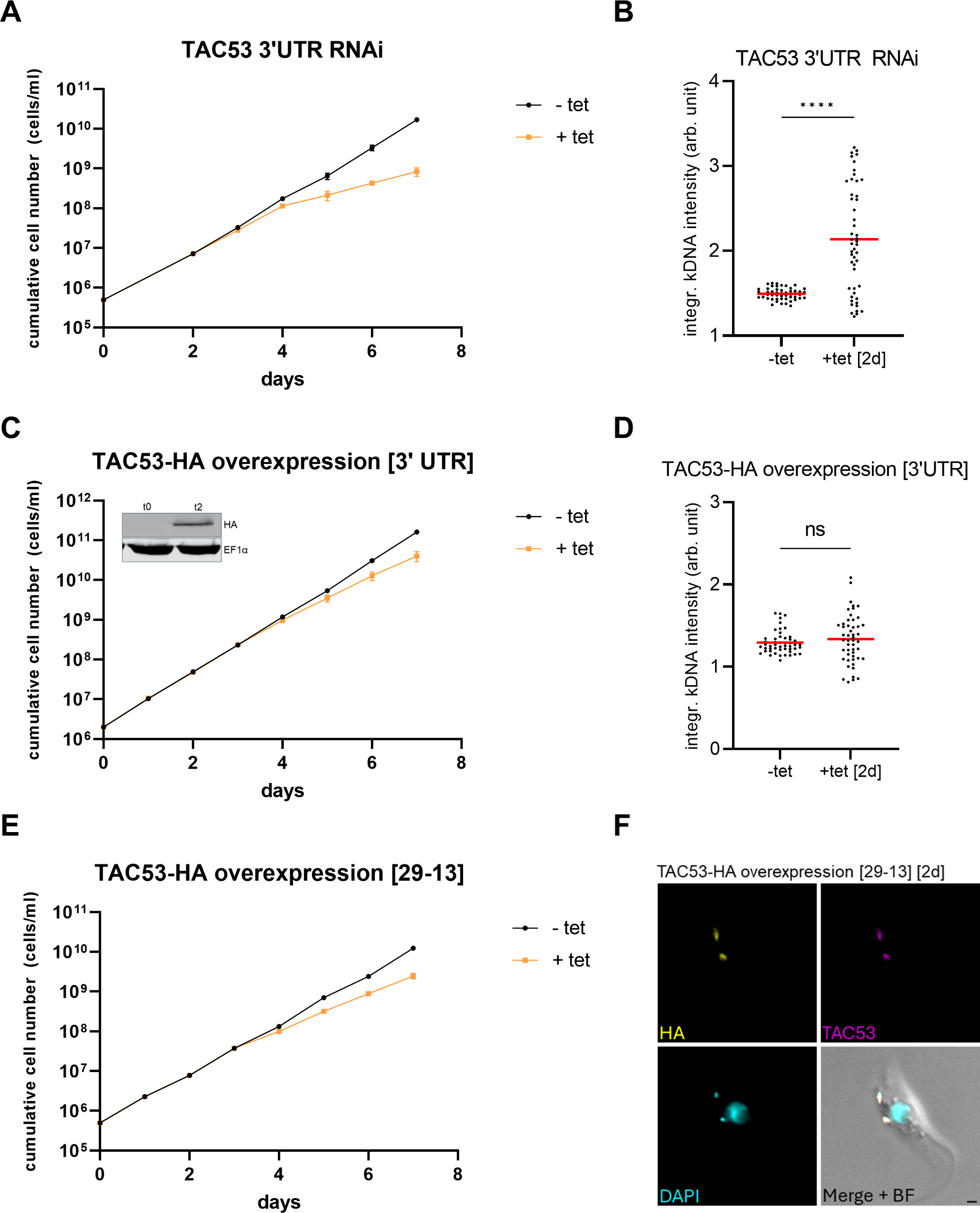

Supplement: S5 Fig — (A) Growth curve of procyclic form cells targeting the 3’ UTR of TAC53. Black line: uninduced cells; orange line: induced cells. (B) Integrated kDNA intensities measured in uninduced and induced [2d] cells from panel A. The red line marks the mean. (C) Growth curve of TAC53-HA expression in the background of the 3’ UTR RNAi (exclusive expression). Black line: uninduced cells; orange line: induced cells. Inset: Western blot showing HA signal after two days of induction, with EF1α as the loading control. (D) Integrated kDNA intensities measured in uninduced and induced [2d] cells from panel C. (E) Growth curve of TAC53-HA overexpression in 29–13 cells. Black line: uninduced cells; orange line: induced cells. (F) Immunofluorescence analysis of TAC53-HA overexpression. The HA tag is shown in yellow, the TAC53 antibody signal is shown in magenta, and DAPI is shown in cyan. BF = Brightfield. Scale bar is 1 μm. (TIF) [file ppat.1013521.s005.tif]

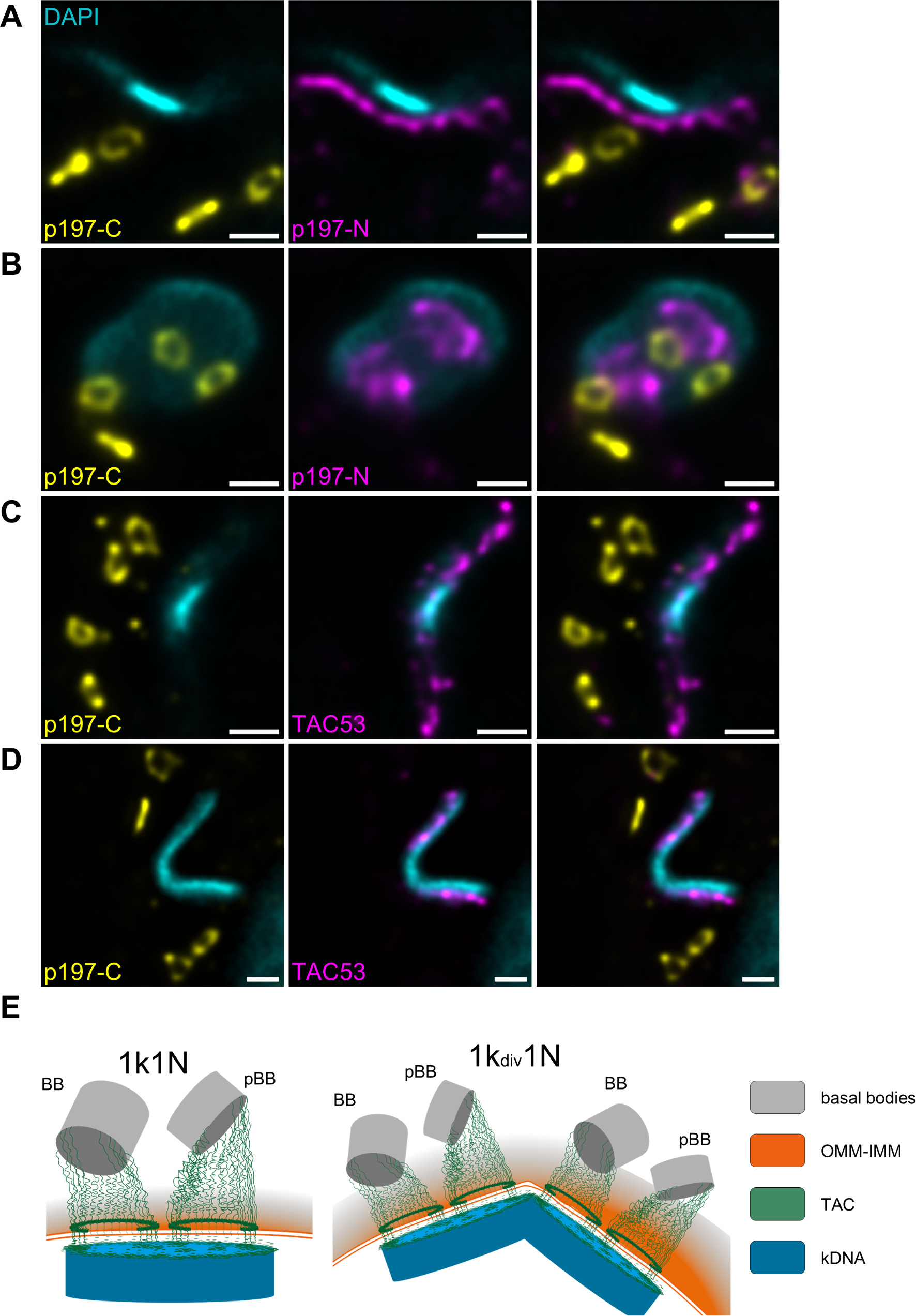

Supplement: S6 Fig — (A) Lateral perspective of replicated kDNAs showing the p197 C- and N-terminus. The C-terminus is shown in yellow, the N-terminus in magenta, and DAPI is shown in cyan. (B) Replicated kDNA shown from an axial perspective with p197 C-terminus in yellow and p197 N-terminus in magenta. DAPI is shown in cyan. (C) (D) Replicated kDNA shown from two different lateral perspectives with p197 C-terminus in yellow, TAC53 in magenta, and DAPI in cyan. Scale bar for A-D is 1 μm. (E) Illustration of the observed structure in expansion microscopy showing pro- and basal bodies (pBB and BB), outer- and inner mitochondrial membranes (OMM-IMM), the TAC, and the kDNA in 1k1N and 1kdiv1N cells. Color coding is found in the figure. (TIF) [file ppat.1013521.s006.tif]

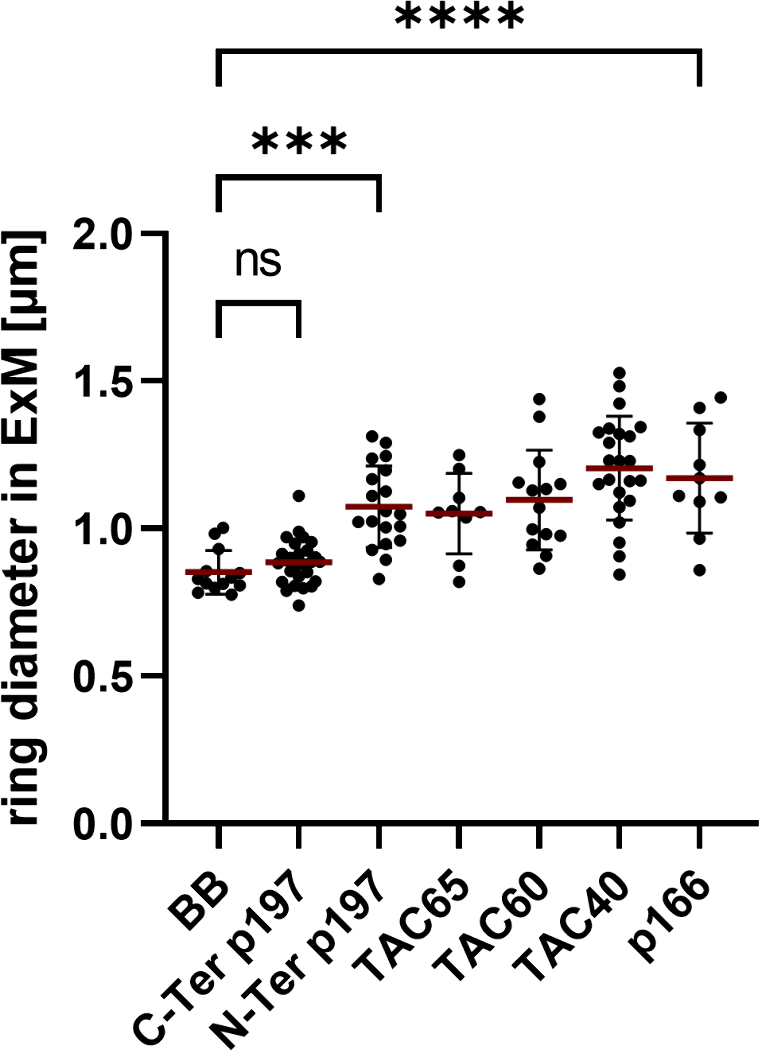

Supplement: S7 Fig — Diameters of ring-like structures were measured from Expansion Microscopy images for the basal body and several TAC proteins, including the C- and N-terminus of p197, TAC65, TAC60, TAC40, and p166. The y-axis shows the measured ring diameters in micrometers (µm), and the x-axis indicates the specific proteins. Each data point represents an individual measurement. Red line marks the mean. Statistical analysis was performed using GraphPad Prism with ordinary one-way ANOVA followed by Bonferroni’s multiple comparison test. (TIF) [file ppat.1013521.s007.tif]

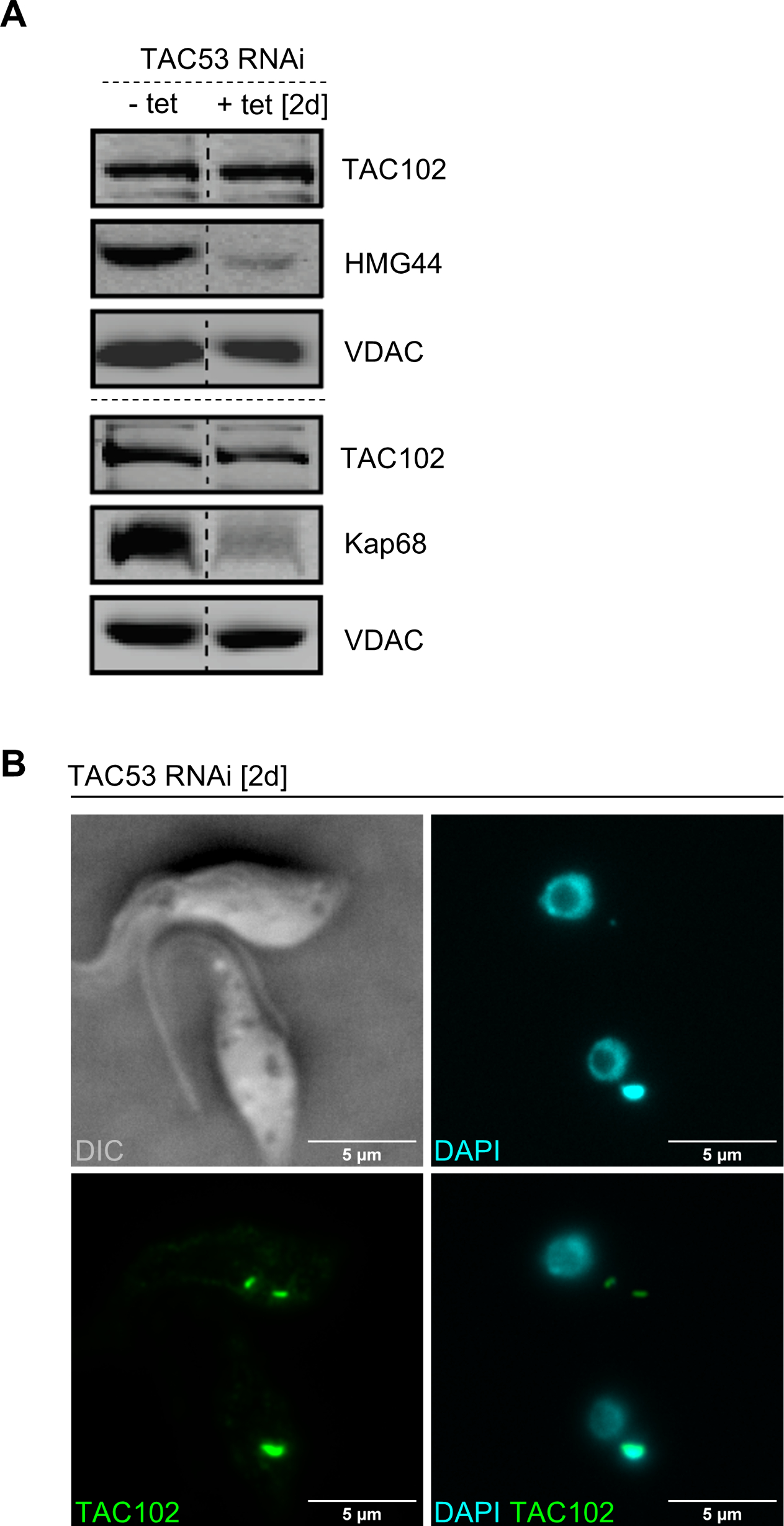

Supplement: S8 Fig — (A) HMG44 and KAP68 are depleted after TAC53 RNAi [2d], while TAC102 remain unchanged. Western Blot analysis of TAC102, HMG44 and KAP68 signals following TAC53 depletion. VDAC serves as a loading control. (B) Immunofluorescence analysis of TAC53-depleted cells [2d], showing TAC102 signals in green, DAPI in cyan, and DIC. Scale bar is 5 μm. (TIF) [file ppat.1013521.s008.tif]

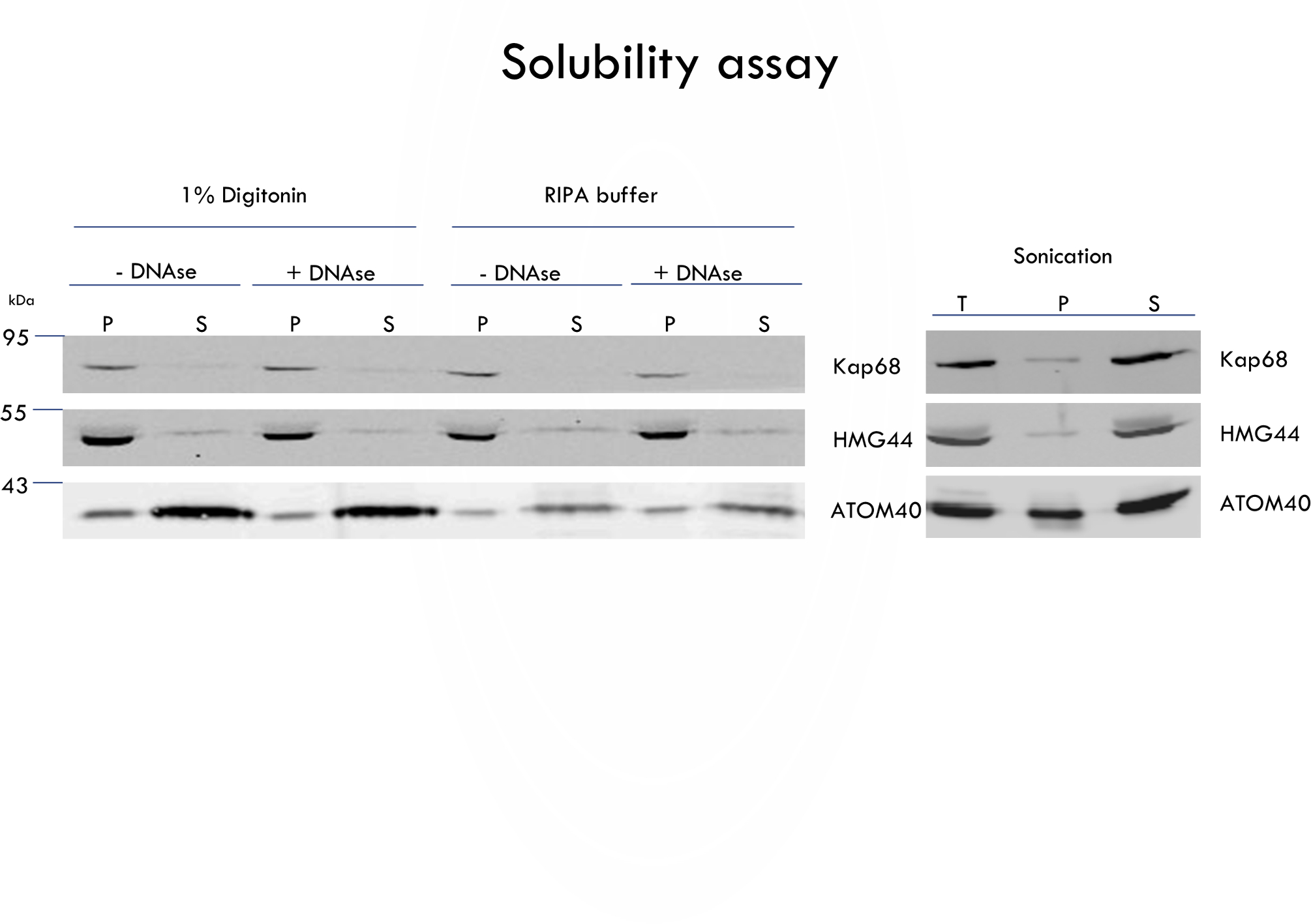

Supplement: S9 Fig — (A) Western blot analysis showing pellet and supernatant fractions for HMG44 and KAP68 after treatment with 1% digitonin or RIPA buffer. Enriched mitochondria were either treated with DNase-I or left untreated. (B) Western blot analysis showing total cells, pellet, and supernatant after sonication. ATOM40 serves as the loading control. (TIF) [file ppat.1013521.s009.tif]

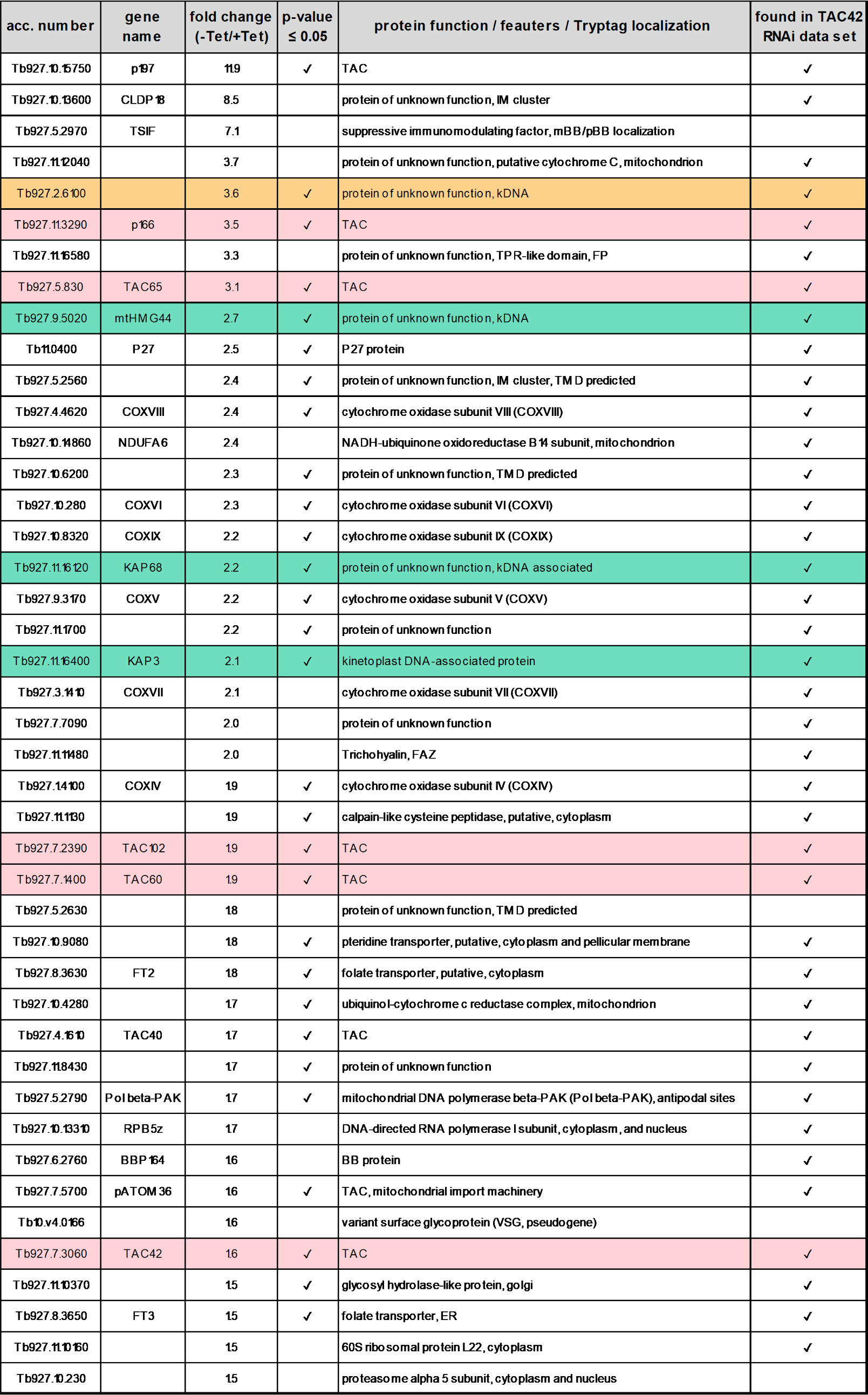

Supplement: S1 Table — Proteins showing >1.5-fold downregulation are included, along with gene name, depletion factor, significance (p ≤ 0.05), TriTrypDB annotation, localization from TrypTag.org, and indication of detection in the TAC42 RNAi dataset. Proteins downregulated >1.5-fold in both datasets are color-coded as in Fig 1. (TIF) [file ppat.1013521.s010.tif]

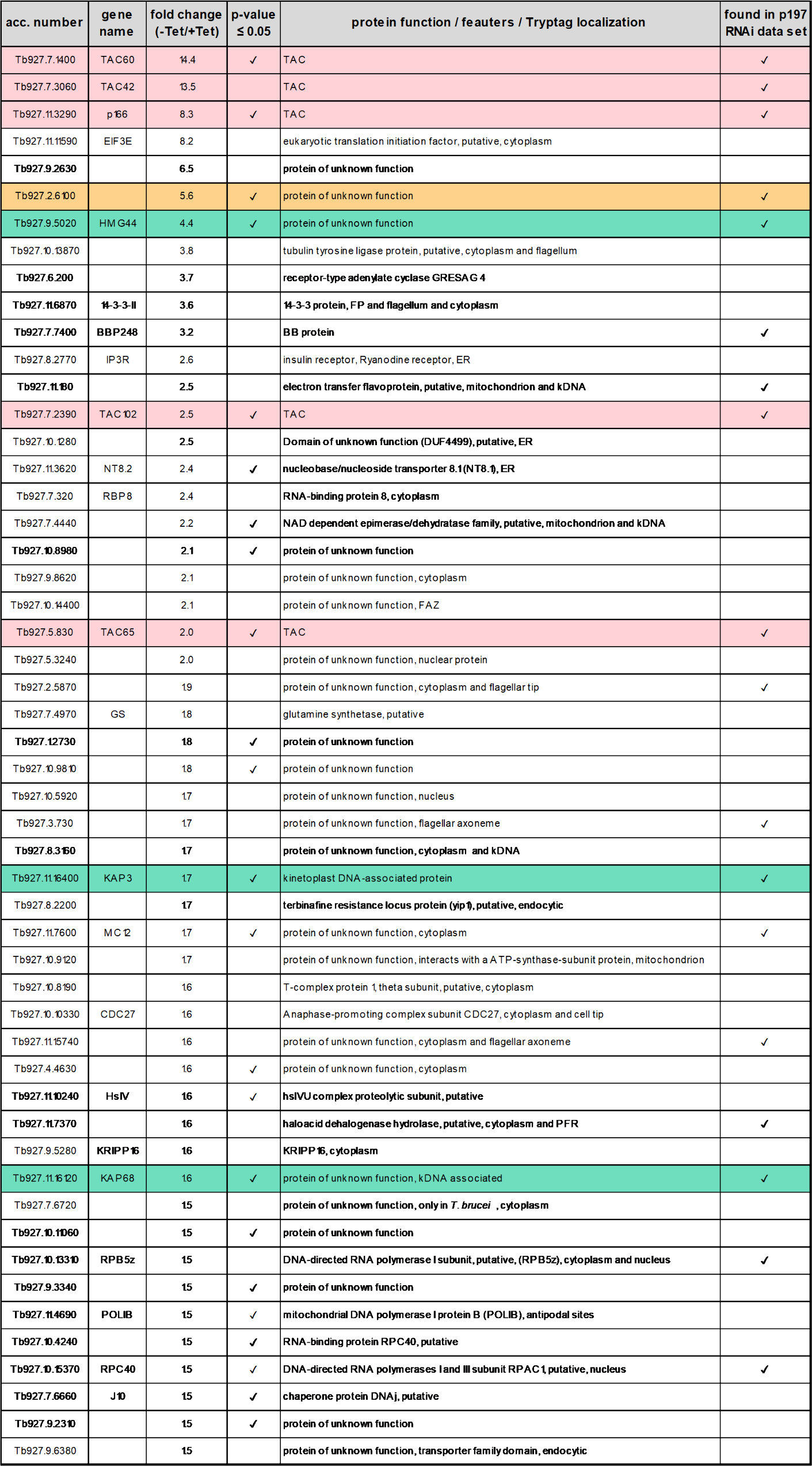

Supplement: S2 Table — Proteins showing >1.5-fold downregulation are included, along with gene name, depletion factor, significance (p ≤ 0.05), TriTrypDB annotation, localization from TrypTag.org, and indication of detection in the p197 RNAi dataset. Proteins downregulated >1.5-fold in both datasets are color-coded as in Fig 1. (TIF) [file ppat.1013521.s011.tif]

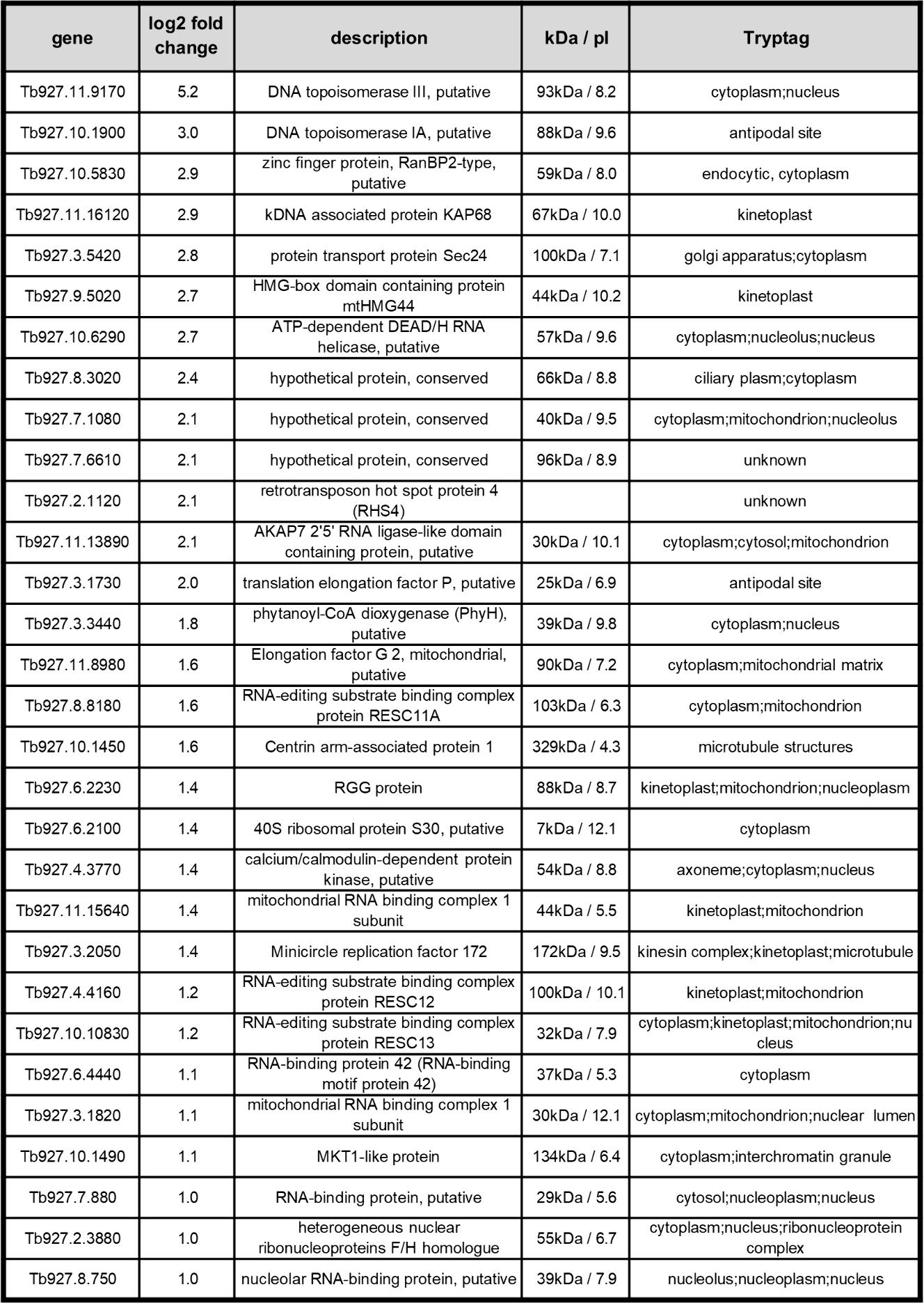

Supplement: S3 Table — All proteins upregulated by more than 2-fold and with a p-value ≤ 0.05 in KAP68HA-tagged versus wild-type (wt) immunoprecipitation are listed. The table includes accession number, log2 fold change, gene description, molecular weight (kDa), isoelectric point (pI), and localization according to TrypTag.org. (TIF) [file ppat.1013521.s012.tif]

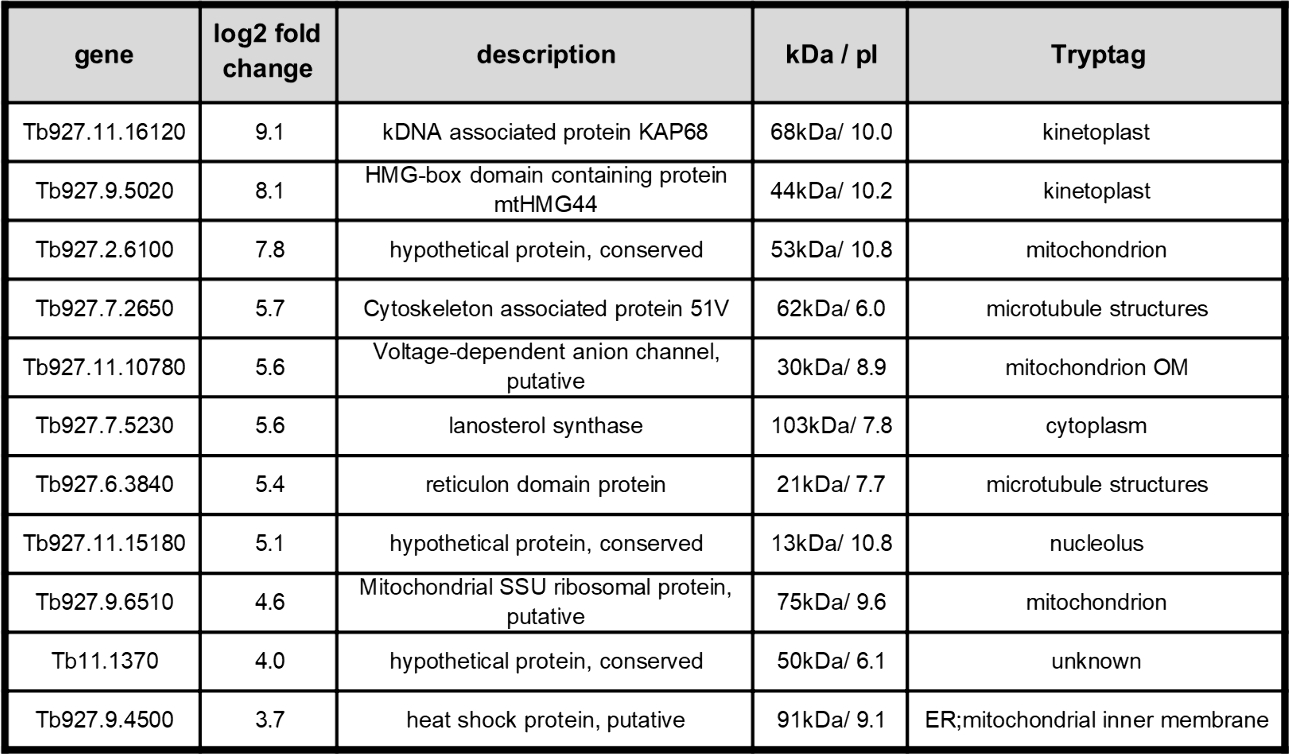

Supplement: S4 Table — All proteins upregulated by more than 2-fold and with a p-value ≤ 0.05 in HMG44myc-tagged versus wild-type (wt) immunoprecipitation are listed. The table includes accession number, log2 fold change, gene description, molecular weight (kDa), isoelectric point (pI), and localization according to TrypTag.org. (TIF) [file ppat.1013521.s013.tif]

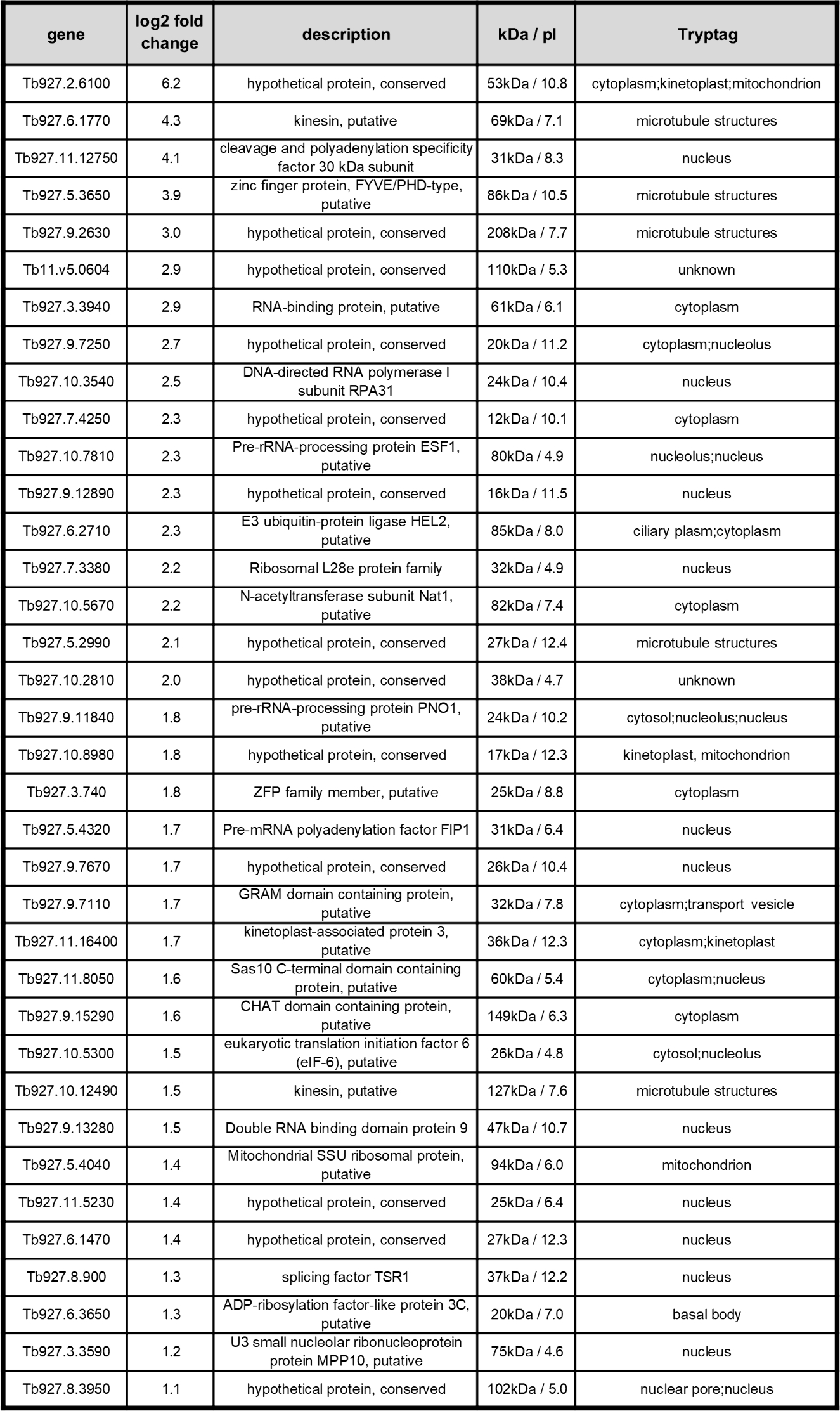

Supplement: S5 Table — All proteins upregulated by more than 2-fold with a p-value ≤ 0.05 in TAC53-HA induced versus uninduced conditions are listed. The table includes accession number, log2 fold change, gene description, molecular weight (kDa), isoelectric point (pI), and localization according to TrypTag.org. (TIF) [file ppat.1013521.s014.tif]

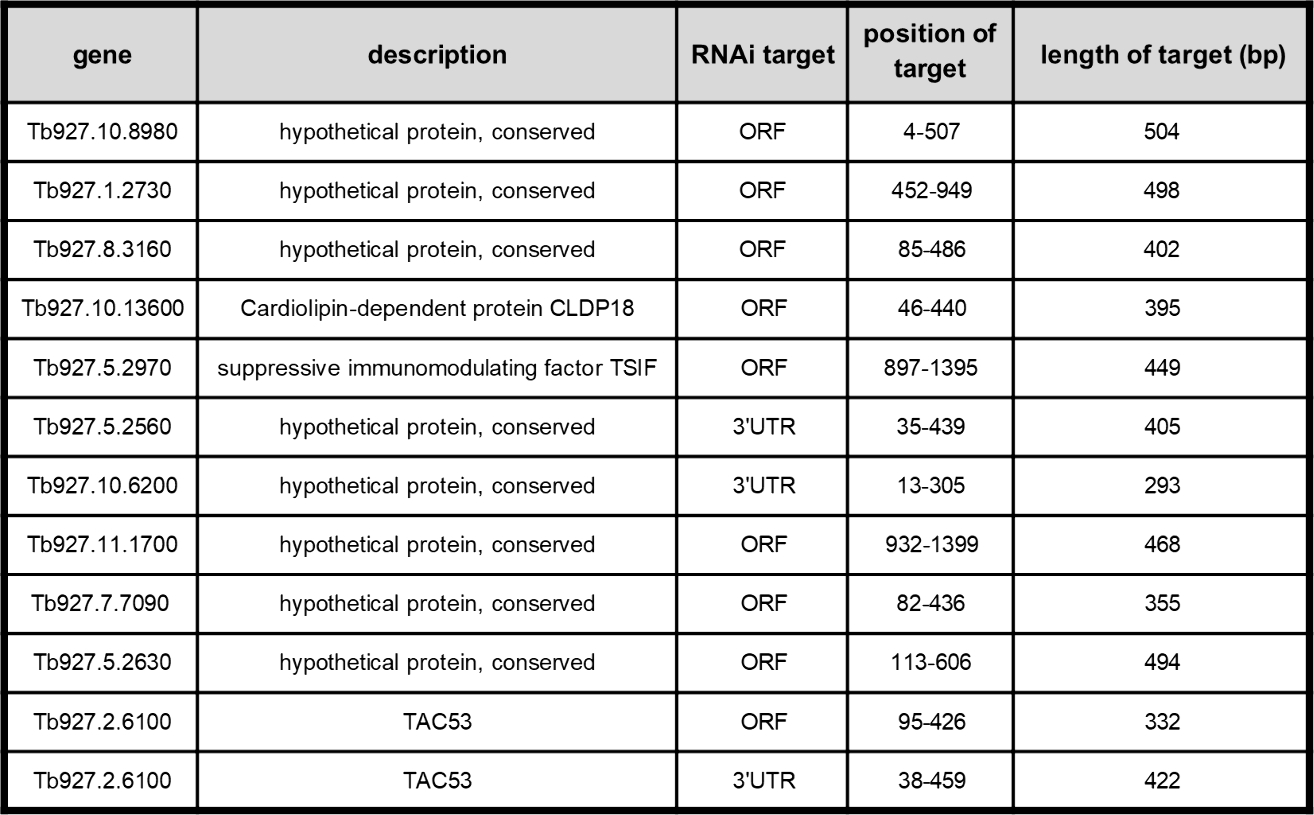

Supplement: S6 Table — The table includes accession number, gene description, RNAi target, position of target, and length of RNAi target. (TIF) [file ppat.1013521.s015.tif]
